# Milk related excipients in medications: concerns with cow’s milk protein allergy

**DOI:** 10.1080/07853890.2021.1896091

**Published:** 2021-09-28

**Authors:** A. Figueiredo, M. Couto, I. M. Costa

**Affiliations:** a Centro de Investigação Interdisciplinar Egas Moniz (CiiEM), Egas Moniz Cooperativa de Ensino Superior, Caparica, Portugal; b PharmSci Lab, Centro de Investigação Interdisciplinar Egas Moniz (CiiEM), Egas Moniz Cooperativa de Ensino Superior, Caparica, Portugal; c José de Mello Saúde, Imunoalergologia, Portugal

## Abstract

**Introduction:**

Food allergy is an increasing health care concern, being cow’s milk protein allergy (CMPA) the most prevalent food allergy in infants and young children (2–7.5%) [1,2]. CMPA symptoms involve mostly skin, gastrointestinal and respiratory reactions. Some exclusively breast-fed infants may also develop CMPA *via* dairy protein transfer through human breast milk [2]. These patients need to strictly avoid all dairy products as well as all traces of milk in all products, including medicines [3]. Milk proteins include serum or whey proteins (α and β-lactoglobulin, albumin serum and immunoglobulins) and caseins. Although not being a protein, lactose (milk sugar) can contaminated with milk proteins residues and it may trigger allergic reactions. This survey aims to examine the composition of some medications commonly consumed by children or during breastfeeding, identifying the drugs with milk related excipients.

**Materials and methods:**

Selection criteria included medicines commercialised in Portugal, containing milk or related compounds, or any of the following constituents: lactose, casein, lactoglobulin, lactalbumin, immunoglobulins, serum albumin, lactoferrin. The full list of excipients has been consulted in the Summary of Drug Characteristics (available in Infarmed site – Portuguese Authority of Medicines and Health Products) [4]. A total of 98 drugs containing the following PI: ibuprofen, paracetamol, iron compounds, bacteria/yeast to replace intestinal flora, desogestrel or amoxicillin, were identified.

**Results:**

24 medicinal products presented lactose as an excipient. In two drugs the excipient found was “cream flavor”. It is noteworthy that all the examined drugs to replace intestinal flora and desogestrel-only pills contain milk related compounds in its composition. Detailed results may be found in Table 1. **Discussion and conclusions:** Several medicines contain lactose, but this excipient can be contaminated with milk proteins [3] and may trigger allergic reactions in CMPA patients, as well as in lactose intolerant individuals. This survey emphasises that health professionals must be aware of which products and ingredients to avoid in these patients.

**Table 1.** Milk related excipients in drug products.

	Analyzed medicines	With milk related excipients
Ibuprofen (oral suspensions, paediatric suppositories and 400 mg tablets)	38	12
Paracetamol (syrups, paediatric suppositories and 1 g tablets)	27	1
Iron compounds (non-injectable formulations)	12	3
Bacteria/yeast to replace intestinal flora	6	6
Desogestrel-only pills	4	4
Amoxicillin (powder for oral suspensions)	11	0
Total	98	26
